# Delayed rupture of right external iliac artery pseudoaneurysm after removal of foreign body from small intestine: a case report

**DOI:** 10.1093/jscr/rjag757

**Published:** 2026-08-31

**Authors:** Lanhui Zeng, Ling Huang

**Affiliations:** Department of Interventional and Vascular Surgery, Affiliated Hospital of Jinggangshan University, No. 1 Quanshuiyan Road, Jizhou District, Ji'an, Jiangxi Province 343009, China; Department of Interventional and Vascular Surgery, Affiliated Hospital of Jinggangshan University, No. 1 Quanshuiyan Road, Jizhou District, Ji'an, Jiangxi Province 343009, China

**Keywords:** small bowel foreign body, pseudoaneurysm, external iliac artery, postoperative complication, case report

## Abstract

Small bowel foreign bodies in adults are rare, and secondary pseudoaneurysm are even rarer. We report a case of a right external iliac artery pseudoaneurysm that occurred one month after removal of a small bowel foreign body. A woman presented with bleeding from a pelvic drainage tube one month after open surgery for small bowel foreign body removal. Preoperative computed tomography (CT) had clearly demonstrated the foreign body in the right pelvic intestine, adjacent to the right external iliac artery. Contrast-enhanced CT at the time of the bleeding showed pelvic hemoperitoneum but did not clearly demonstrate a pseudoaneurysm. Digital subtraction angiography revealed the pseudoaneurysm, which was successfully treated by implantation of a self-expanding covered stent. The patient recovered well and was discharged. Although a pseudoaneurysm triggered by a small bowel foreign body is rare, this case underscores the importance of postoperative monitoring, particularly attention to changes in drainage fluid and timely imaging examinations for diagnosis and intervention.

## Introduction

Small bowel foreign bodies are non-physiological objects that enter the small intestine through the oral cavity or other routes, often leading to intestinal obstruction, injury, or infection, with a relatively low incidence in adults [1]. A pseudoaneurysm is a hematoma formed around an artery after arterial wall rupture, lacking a complete arterial wall. Pseudoaneurysms are relatively common among complications following abdominal surgery, particularly those related to vascular injury [2–4]. Abdominal pain, nausea, vomiting, and obstruction are typical of small bowel foreign bodies, yet nonspecific symptoms impede early diagnosis [5–7]. Correspondingly, pseudoaneurysms may present as persistent abdominal or pelvic bleeding, particularly after rupture, which may be accompanied by hypotension and shock, complicating the diagnosis further [8, 9]. This case reports a pseudoaneurysm of the right external iliac artery occurring after small bowel foreign body removal. Through timely intervention, the patient ultimately recovered and was discharged, providing valuable experience for the management of similar cases.

## Case report

**Figure 1:**
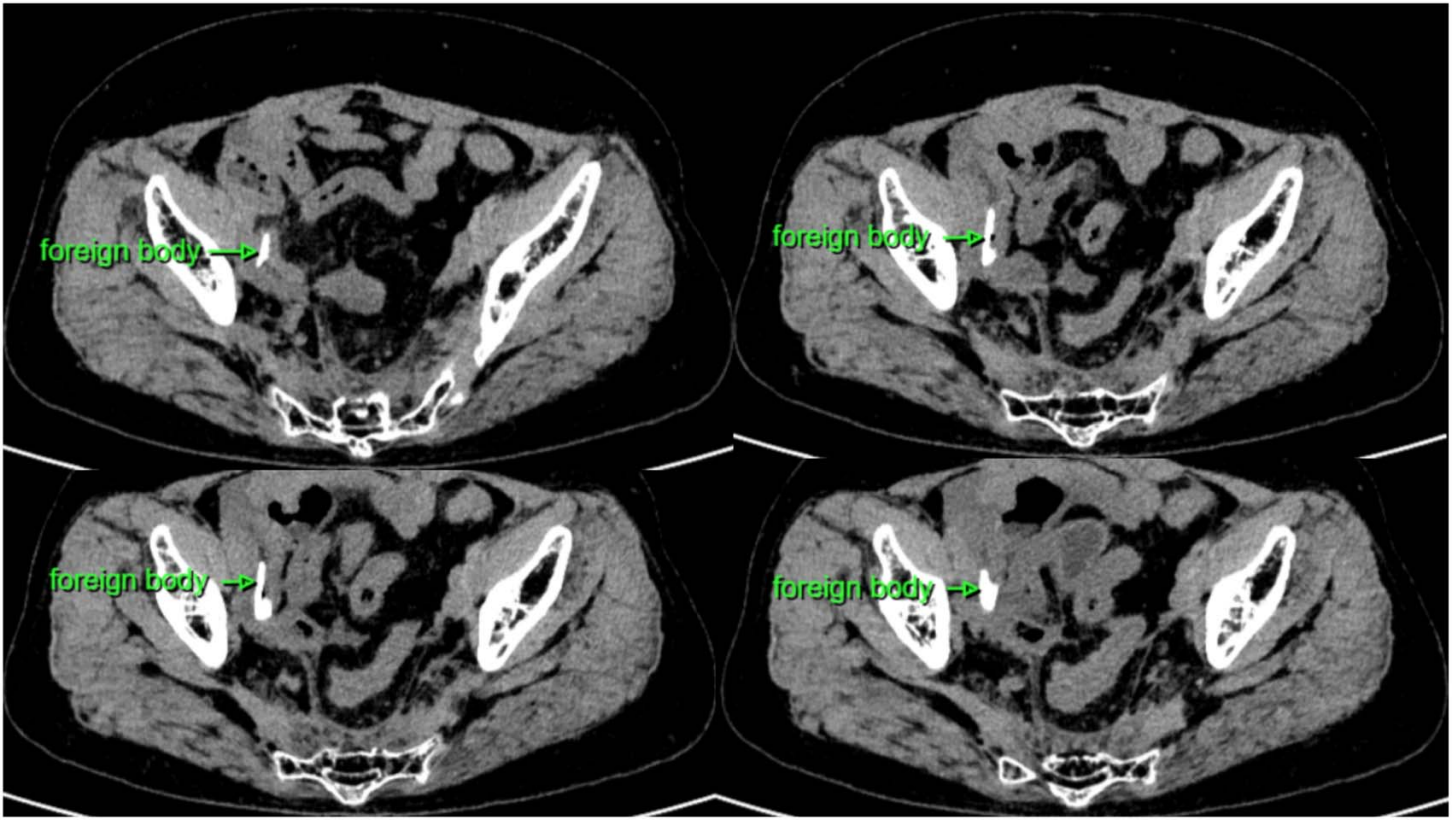
Preoperative abdominal CT (plain scan) obtained prior to open surgical removal of the foreign body shows a strip-like hyperdense foreign body (arrow) in the right pelvic small intestine, with adjacent mesenteric haziness indicating inflammatory changes.

A 54-year-old woman was admitted to our hospital after experiencing bleeding from the pelvic drainage tube for half a day, discovered one month after surgery for small bowel foreign body removal. One month prior, the patient had visited a local hospital due to a small bowel foreign body; abdominal computed tomography (CT) plain scan showed a strip-like dense shadow in the right pelvic cavity intestinal canal, suspected to be a foreign body. The imaging clearly demonstrated that the foreign body was located in the small intestine of the right pelvic cavity, with its position closely adjacent to the right external iliac artery (Fig. 1). Open surgery was performed, during which severe adhesions were noted in the right lower quadrant, making the dissection challenging. Due to the severe pelvic adhesions, no dissection around the vessels was attempted, and the vessels were not injured. Intraoperatively, the small intestine was found to have been perforated by the foreign body. Partial resection of the small intestine, removal of the foreign body, and adhesion release were performed, and a pelvic drainage tube was left in place postoperatively. The patient was discharged after an uneventful initial recovery, but the drain was maintained due to persistent purulent discharge, suggesting ongoing local inflammation.

**Figure 2:**
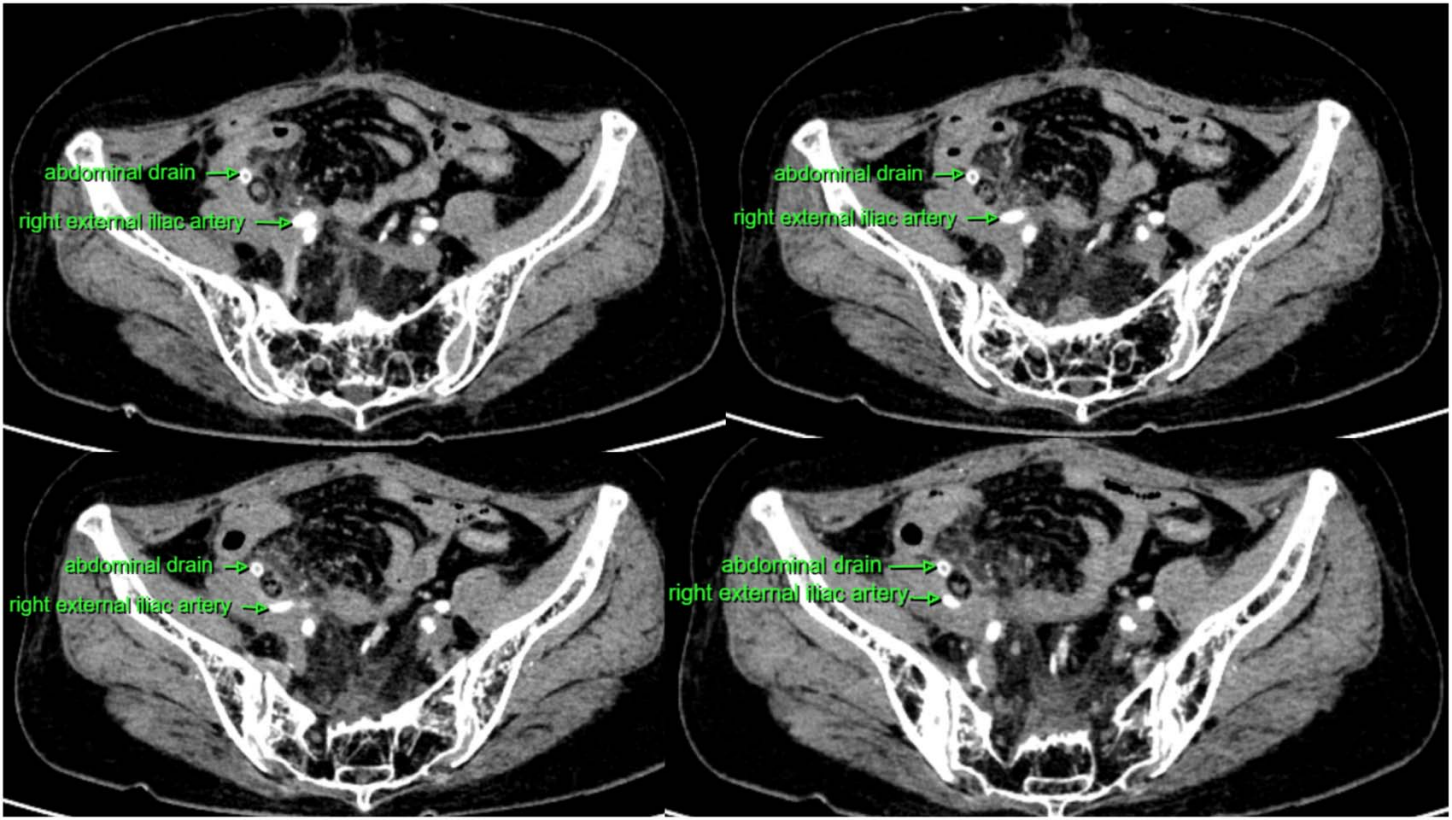
Contrast-enhanced CT (arterial phase) at the time of active bleeding, one month after open surgical removal of the foreign body. The arrows mark the right external iliac artery and the pelvic drainage tube, respectively. No definite pseudoaneurysm or active extravasation is identified.

**Figure 3:**
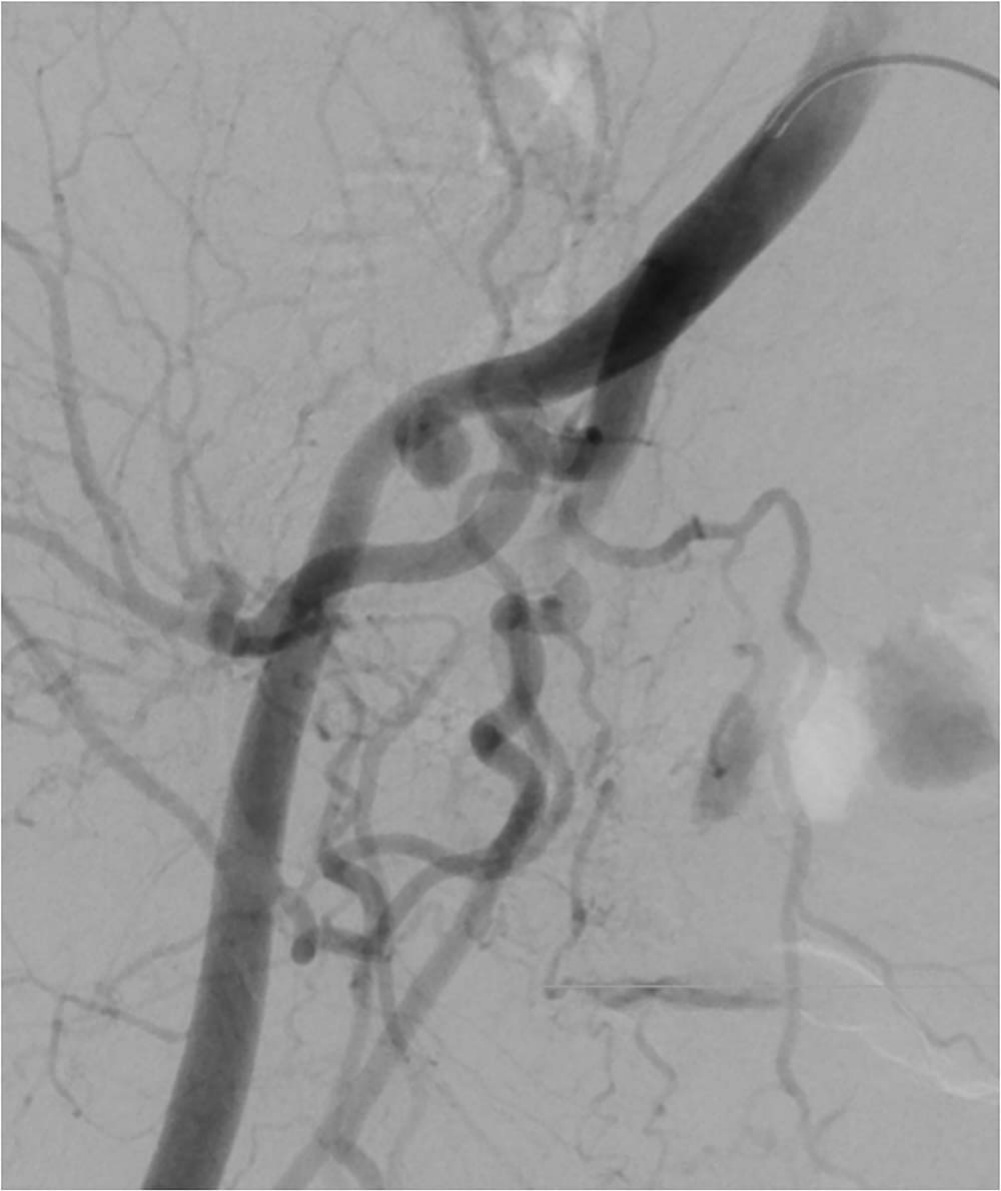
Pre-interventional angiography showing a pseudoaneurysm located in the right external iliac artery.

**Figure 4:**
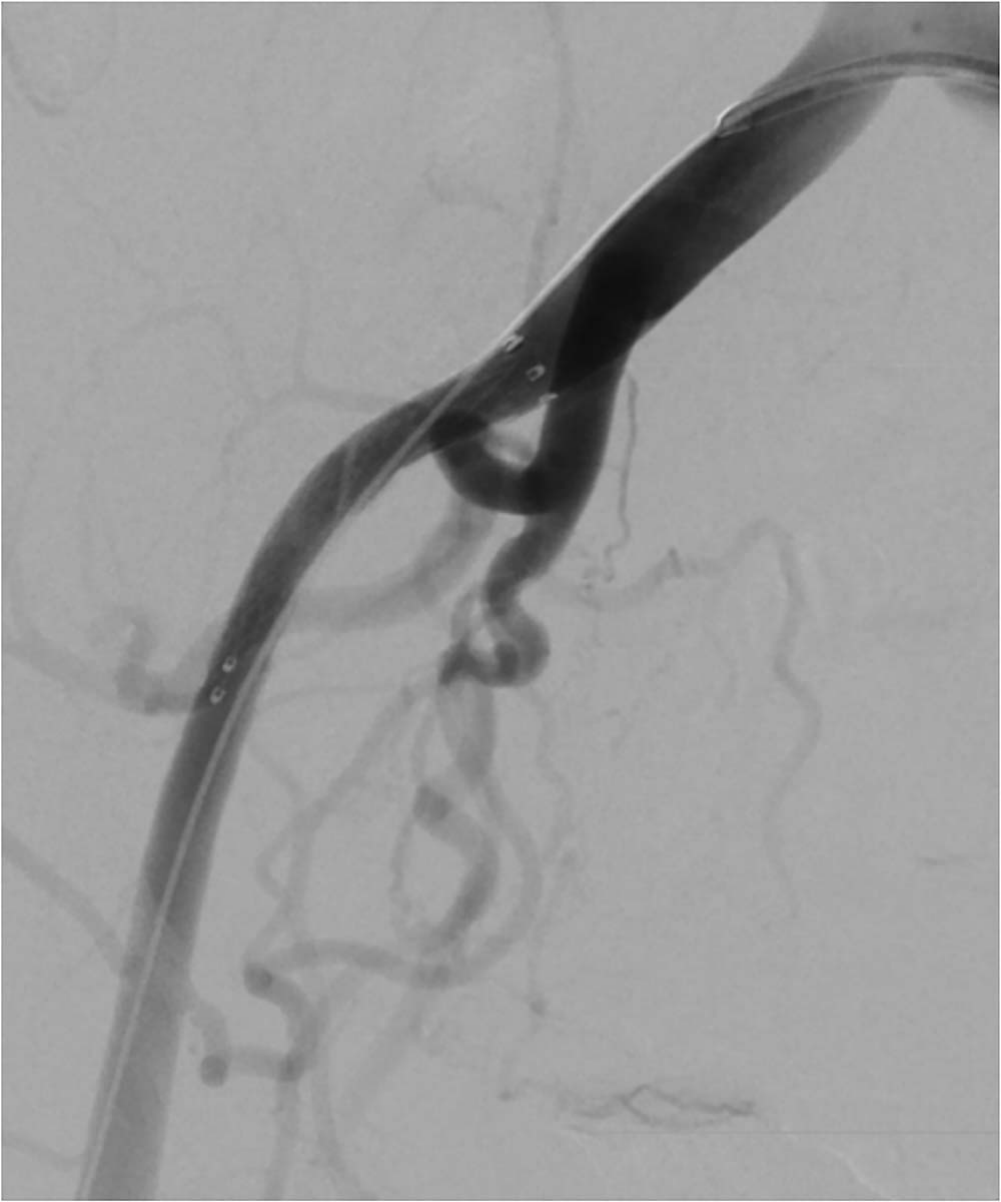
Implantation of a self-expanding covered stent (8 × 40 mm) was performed, followed by angiography, which showed complete isolation of the pseudoaneurysm and patency of the parent artery lumen.

On the evening of 7 September 2025, the drainage tube output 200 mL of blood, but no special treatment was administered. The next morning, the drainage tube had output an additional 300 ml of blood. Contrast-enhanced CT was then performed, which did not reveal any obvious pseudoaneurysm or bleeding site (Fig. 2). Digital subtraction angiography (DSA) performed at the local hospital revealed a pseudoaneurysm of the right external iliac artery, measuring ~2 mm in diameter. The patient was subsequently transferred to our hospital for further evaluation. Repeat angiography demonstrated significant enlargement of the pseudoaneurysm, with a risk of rupture and bleeding at any time (Fig. 3). A self-expanding covered stent (8  × 40 mm) was implanted to stabilize the vascular structure. Follow-up angiography confirmed that the pseudoaneurysm had disappeared (Fig. 4). Postoperatively, drainage ceased, and the drainage tube was removed after 3 days of observation. One week post-procedure, the patient had recovered well and was discharged.

The pseudoaneurysm in this patient may have resulted from a combination of factors. The foreign body’s location in the right pelvic intestine was anatomically adjacent to the right external iliac artery, as confirmed by preoperative CT (Fig. 1), and the severe local inflammation and adhesions might have predisposed the vessel wall to injury. Although no bleeding occurred during the foreign body removal surgery, delayed rupture of the pseudoaneurysm manifested one month postoperatively. Notably, the initial contrast-enhanced CT at the time of bleeding showed only hemoperitoneum without clear evidence of a pseudoaneurysm (Fig. 2), highlighting the diagnostic challenge and the critical role of DSA in establishing the final diagnosis. After timely interventional treatment, the patient ultimately recovered.

## Discussion

The occurrence of pseudoaneurysm after small bowel foreign body surgery carries significant clinical implications, particularly regarding the potential for delayed diagnosis. Although pseudoaneurysm caused by small bowel foreign bodies is rare, similar cases reported in the literature provide useful context. In one study, a patient developed a pseudoaneurysm of the external iliac artery after abdominal surgery, ultimately leading to hemorrhagic shock [10]. In another case involving ovarian cancer surgery, the patient experienced lower abdominal bleeding 20 days postoperatively, diagnosed as a pseudoaneurysm through imaging [11]. These cases highlight the need for heightened vigilance regarding postoperative bleeding, especially after complex surgeries.

The formation mechanism of pseudoaneurysm in this setting warrants consideration. After penetrating the intestinal wall, foreign bodies may incite a severe local inflammatory response and adhesion formation. In our case, the foreign body's proximity to the right external iliac artery on CT suggests local inflammation may weaken the vessel (Fig. 1), increasing susceptibility to injury. CT during hemorrhage revealed only hemoperitoneum without pseudoaneurysm (Fig. 2), highlighting CT's limitation and the need for DSA when suspicion persists. The role of imaging in prompt detection of pseudoaneurysms, particularly DSA, has been emphasized in the literature [12]. “Several lessons emerge: postoperative bleeding requires vigilance for vascular injury, particularly after complex procedures. Pseudoaneurysm formation involves local damage and inflammation, emphasizing early recognition and dynamic monitoring [13]. In our case, negative or equivocal CT with persistent bleeding led to prompt DSA, which confirmed the pseudoaneurysm and allowed endovascular treatment.”

Regarding the mechanism, the penetration of a small bowel foreign body may directly damage surrounding vessels, a mechanism supported by the literature [14]. The timing of intervention is equally important, as early intervention can effectively reduce complications [15]. Anatomical variation and postoperative inflammatory changes may further contribute to vascular vulnerability.

Despite the clinical value of this case, certain limitations exist. The uniqueness of an individual case may limit general applicability, and future research should focus on larger sample sizes to validate these findings. Although intraoperative photographs were lacking, serial CT before and during bleeding provided indirect evidence of progression; however, negative CT cannot exclude vascular bleeding, and DSA remains the gold standard, warranting further investigation into the mechanisms and their association with foreign bodies. In summary, the management of pseudoaneurysm following small bowel foreign body surgery underscores the importance of early identification, dynamic monitoring, and timely intervention, with DSA playing a pivotal role when CT findings are inconclusive.

## Data Availability

Not applicable.
